# Complete Genome Sequences of Two Chikungunya Viruses Imported into China

**DOI:** 10.1128/genomeA.00480-18

**Published:** 2018-06-28

**Authors:** Xiaomin Zhang, Yalan Huang, Miao Wang, Fan Yang, Jinquan Cheng, Chengsong Wan, Renli Zhang

**Affiliations:** a Shenzhen Center for Disease Control and Prevention, Shenzhen, China; b School of Public Health and Tropical Medicine, Southern Medical University, Guangzhou, China

## Abstract

Here, we isolated two chikungunya virus strains (SZ1050 and SZ1239) from patients infected with chikungunya virus who were returning to China from India and Indonesia, respectively. Strain SZ1050 was classed in the Indian Ocean lineage, and strain SZ1239 belonged to the Asian lineage. No A226V mutation in the envelope protein E1 was found in either strain.

## Abstract (precis)

Here, we isolated two chikungunya virus strains (SZ1050 and SZ1239) from patients infected with chikungunya virus who were returning to China from India and Indonesia, respectively. Strain SZ1050 was classed in the Indian Ocean lineage, and strain SZ1239 belonged to the Asian lineage.

## GENOME ANNOUNCEMENT

Chikungunya virus (CHIKV) is an arthropod-borne alphavirus belonging to the family Togaviridae. CHIKV is the causative agent of chikungunya fever (CHIKF), characterized by high fever, rash, arthralgia, and even sometimes death (1). Recently, CHIKV’s reemergence in multiple regions has been considered a global threat to public health (2). There are no effective vaccines or treatments for CHIKV infection (3). Thus, there is an urgent need for a better understanding of CHIKV pathogenesis and epidemics.

In October 2010, a patient back from New Delhi, India, had a fever with chest pain and arthralgia. He was diagnosed with a chikungunya virus infection by real-time fluorescent quantitative PCR. The virus was then isolated in C6/36 cells from his serum sample and named strain SZ1050 (4). Another CHIKV strain, SZ1239, was isolated from a 26-year-old female patient who had traveled back from Indonesia in July 2012 (5). Both virus strains were passaged in a BHK-21 cell line. Viral RNA was extracted by using a QIAamp viral RNA minikit (Qiagen, Germany) and reverse transcribed into cDNA by ReverTra Ace qPCR RT kit (Toyobo, Japan). Primers were designed according to the sequence of Ross (GenBank accession no. AF490259). PCR products were sequenced by Sanger sequencing to determine the open reading frames (ORFs). The sequences of the 5′ untranslated region (UTR) and 3′ UTR were investigated by using a SMARTer rapid amplification of cDNA ends (RACE) 5′/3′ kit (TaKaRa, Japan).

The complete genome sequence of SZ1050 was determined to be 11,844 bp, including two ORFs flanked by the 5′ UTR (86 nucleotides [nt]) and 3′ UTR (521 nt). One ORF was responsible for translation of the nonstructural polyprotein (7,422 nt, 2,474 amino acids [aa]), whereas the second ORF contained the structural polyprotein (3,744 nt, 1,248 aa). There are three repeated sequence elements (RSEs) in the 3′ UTR of SZ1050, similar to the RSEs in Ross (6). The complete genome sequence of SZ1239 was determined to be 12,000 bp, including two ORFs flanked with a 5′ UTR and 3′ UTR. The nonstructural protein (nsP) of SZ1239 consisted of 2,467 amino acids, including nsP1 (535 aa), nsP2 (798 aa), nsP3 (523 aa), and nsP4 (611 aa). Of note, compared to the amino acid sequence of Ross, a 7-aa deletion was observed in the nsP3 of SZ1239, which was also found in other studies (GenBank accession no. HE806461, KM673291, and KX097982) (7–9). In addition, in the 3′ UTR of SZ1239, only two RSEs were observed, similar to those of the three CHIKV isolates mentioned above (7–9). No A226V mutation in the envelope protein E1 was found in either strain. Phylogenetic analysis indicated that SZ1050 was classed into the Indian Ocean lineage, whereas SZ1239 belonged to the Asian lineage. Whether the deletions in the nsP3 and 3′ UTR contribute to the persistence of SZ1239 remains unclear.

### Accession number(s).

The complete genome sequences of the two CHIKV strains in this study have been deposited in GenBank under the accession no. MG664850 (SZ1050) and MG664851 (SZ1239).
